# Defining the molecular pathology and consequent phenotypes in Egyptian HB patients

**DOI:** 10.1186/s43141-021-00165-8

**Published:** 2021-05-17

**Authors:** Ghada Y. El-Kamah, Rehab M. Mosaad, Mohamed B. Taher, Khalda S. Amr

**Affiliations:** 1 Clinical Genetics Department, Human Genetics and Genome Research division (HGGR), National Research Centre (NRC), Cairo, Egypt; 2 Molecular Genetics and Enzymology Department, HGGR, NRC, El Buhouth St., Dokki, Cairo, Egypt; 3 Medical Molecular Genetics, HGGR, NRC, Cairo, Egypt

**Keywords:** Hemophilia B, Factor IX gene, Nonsense mutations, Sequencing

## Abstract

**Background:**

Hemophilia B (HB) (also known as Christmas disease) is a rare X-linked recessive disorder characterized by spontaneous or prolonged hemorrhages caused by mutations in Factor 9 (*F9*) gene leading to deficient or defective coagulation *F9*. Our study aimed at identifying the causative mutations within a sample of HB Egyptian patients. The present study comprised clinical data of eleven HB patients descending from six unrelated families and a seventh family including a carrier mother with a history of deceased HB sibling. Sequencing of *F9* gene was performed.

**Results:**

The study revealed four mutations; two missense NM_000133.3:c.676C>G, (P.Arg226Gly) and NM_000133.3:c.1305T>G, (p.Cys435Trp), and two nonsense mutations NM_000133.3:c.880C>T, (p.Arg294*) and NM_000133.3:c.1150C>T, (p.Arg384*), identified mutations spanned exons 6 and 8 of which a total of three mutations are located in hotspot exon 8 of *F9* gene.

**Conclusions:**

Reviewing the literature, this is the first molecular analysis of *F9* gene in HB Egyptian patients. Consistent genotype/phenotypic severity correlation could be concluded, helping proper genetic counseling and prenatal decision taking.

## Background

Hemophilia-B (HB, OMIM 306900) is the second most common form of hemophilia after hemophilia-A accounting for 15-20% of hemophilia affection. HB incidence is estimated as one in 30,000 live male births worldwide with rare reports of affected females [1]. However, reports vary widely, and the prevalence of hemophilia B per 100,000 males in 103 countries ranged from 0.01 (Nigeria) to 8.07 (Ireland). In 2019, the World Federation of Hemophilia (WFH), in its global survey in Egyptian population (98,423,595) estimated the number of affected patients with hemophilia as 6028 [2].

Based on the plasma levels of *F9*, HB patients are clinically classified as severe (<1%), moderate (1-5%), and mild (5-40%). Severely affected males suffer from frequent spontaneous bleeding mainly in joints and muscles while mild or moderate cases experience excessive bleeding only upon trauma [3].

Hemophilia-B is caused by a wide variety of heterogeneous and infrequent mutations in *F9* gene. The *F9* gene (OMIM 300746) located on the Xq27.1 is 34 kb in length encompassing 8 exons encoding 2.8 kb mRNA. Full length *F9* gene contains 451 amino acid residues and includes a signal peptide (1–28 residues), a propertied (29–46) domain Gla enriched with glutamate (47–92), two EGF-like domain EGF1 (93–129) and EGF2 (130–171), a linker (172–191), an activation peptide (192–226), and SP domain of serine protease (227–451) [4, 5].

Underlying *F9* gene mutations is responsible for the phenotypic severity and bleeding tendency. A wide spectrum of *F9* gene mutations causing HB has been identified in all regions of the gene. The most common mutation type is point mutations which account for about 73%, followed by deletion mutations 16.3%, and, in minor proportion; insertions, duplications, small indels, and large rearrangements. More than 3940 unique mutations have been reported so far in HGMD [6]. Most mutations within *F9* gene can be detected by direct DNA sequencing; however, for large gross deletions, the multiple ligation—dependent probe amplification (MLPA) assay could be employed [1].

This study aims at the identification of pathogenic mutations verified by gene screening and describing the clinical outcomes from variants identified in a sample of Egyptian HB patients referred to the Hereditary Blood Disorders clinic, (NRC) Egypt, and develop a first step in building knowledge about the molecular basis of this disease in Egyptian patients ultimately helping to provide proper genetic counseling.

## Methods

The Institutional Review Board NRC according to the World Medical Association Declaration of Helsinki approved the current research. Eleven HB male patients descending from six unrelated pedigrees and a seventh family including a carrier mother with a history of deceased HB patient were recruited from the Hereditary Blood Disorders clinic, NRC. Patients’ ages ranged from 1 to 20 years old and their ages at disease onset ranged from 22 days-3 years. They were all descending from non-consanguineous families. Patients were included based on the presence of a history of bleeding and laboratory findings including prolonged activated partial thromboplastin time (aPTT), normal thromboplastin time (PT), and deficient F9. Patients were subjected to detailed medical history recording including three-generation pedigree analyses, demographic data, initial complaint, age at onset, history of present illness, and disease progression, coupled with thorough clinical evaluation with emphasis on hemarthrosis. Patients were then phenotypically classified according to bleeding episodes and the level of F9 activity into one mild (>5%) and two moderate (1–5%) HB patients with history of severe bleeding following trauma, and eight severe (<1%) HB patients presenting with history of spontaneous joints and/or muscles bleeding episodes. Written informed consents were obtained from all participants and/or legal guardians. Peripheral blood samples (3 ml) were collected in EDTA tubes from all recruited patients and their available family members for genomic DNA extraction.

### Mutation analysis

Genomic DNA was extracted from 2 ml peripheral blood of affected HB patients and their available family members using “DNA Mini kit Quiagen” following the manufacturer’s instructions and quantification of the isolated DNA was performed by spectrometry in a “Nanodrop 2000.”

Eight exons of *F9* gene were amplified by polymerase chain reaction (PCR) of primers designed by ExonPrimer SOFTWARE. The coding regions and exon/intron boundaries of approximately 50 bp sequence were investigated to recognize any splice site variants as well. The PCR products were purified using Exo-SAP PCR purification kit (Fermentas, Germany) and both directions were sequenced using BigDye Terminator v3.1 Cycle Sequencing Kit and analyzed on the ABI Prism 3500 Genetic Analyzer (Applied Biosystems) according to manufacturer’s instructions.

The sequence data of *F9* gene was compared with reference genomic and cDNA sequence of the gene nucleotide numbering based on the Genebank accession numbers IX (F9), RefSeqGen LRG 556 on chromosome X, NM_000133.3 and NP_000124.1., with c.1 denoting the first position of the translation start codon obtained from National Center for Biotechnology Information (NCBI) database. Mutation nomenclature was given according to Genetic Variations approved by the Human Genome Variation Society (HGVS) (http://www.hgvs.org). Results were examined using Ensembl genome browser (https://www.ensembl.org/index.html).

To ascertain whether a variant was novel or not, all of the mutations in this study were compared to the international *F9* gene databases (http://www.factorix.org/; https://www.cdc.gov/ncbddd/hemophilia/champs; http://www.hemobase.com/).

## Results

In this study, eleven HB male patients descending from six unrelated pedigrees were investigated. Patients’ ages ranged from 1 to 20 years old and their ages at disease onset ranged from 22 days-3 years. They were all descending from non-consanguineous families residing in different governorates in Egypt. According to *F9* activity, eight HB cases were phenotypically classified as severe, two as moderate, and one mild. Family 7 presented with a history of previous death of HB sibling with severe phenotype.

**Fig. 1:**
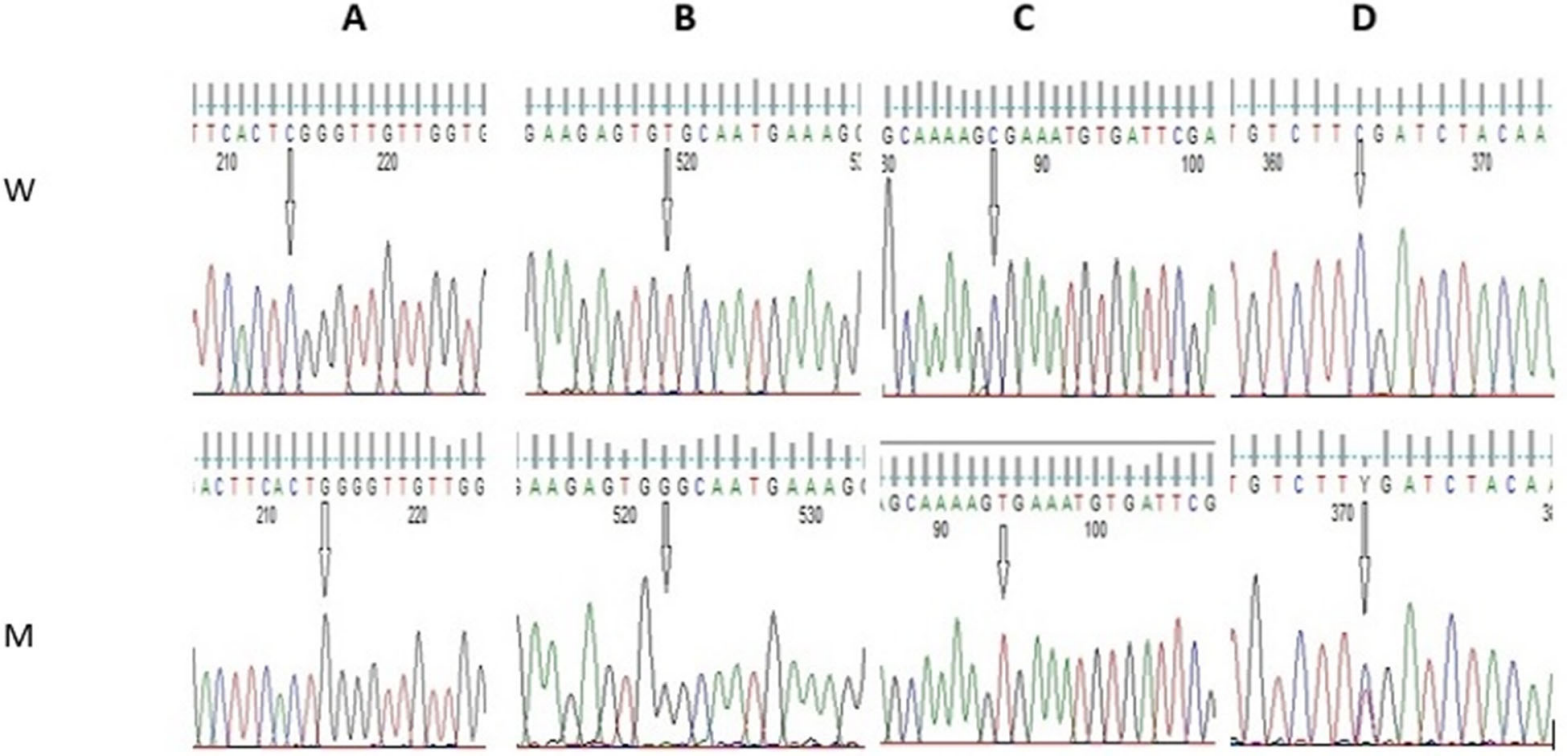
Partial nucleotide sequence of F9 gene. **a** Missense mutation c.676C>G in exon 6 identified in patients no. 1 and 2. **b** Missense mutation c.1305T>G in exon 8 identified in patient no. 3. **c** Nonsense mutation c.880C>T in exon 8 identified in families no. 4, 5, 6. **d** Nonsense mutation c.1150C>T in exon 8 identified in carrier mother family no. 7 (W for wild and M for mutant)

Mutational analysis of the coding region of the *F9* gene identified biallelic pathogenic mutations in all our HB patients (Table 1). Two HB patients 1 and 2 harbored homozygous missense mutation, namely (NM_000133.3: c.676C>G; NP_000124.1: p. Arg226Gly) located in exon six (Fig. 1a).

**Table 1:** Characterization of the variants identified in the families with HB

Patients ID	Nucleotide change NM_000133.3	Location	Amino acid change NP_000124.1	Mutation effect	Severity	Domain	Citation
HB-1	c.676 C>G	Exon 6	p.Arg226Gly	Missense	Moderate	Activation peptide	6
HB-2	c.676 C>G	Exon 6	p.Arg226Gly	Missense	Moderate	Activation peptide	6
HB-3	c.1305 T>G	Exon 8	p.Cys435Trp	Missense	Mild	Serine protease	2
HB-4	c.880 C>T	Exon 8	p. Arg294*	Nonsense	Severe	Serine protease	70
HB-4.1	c.880 C>T	Exon 8	p. Arg294*	Nonsense	Severe	Serine protease	70
HB-4.2	c.880 C>T	Exon 8	p. Arg294*	Nonsense	Severe	Serine protease	70
HB-4.3	c.880 C>T	Exon 8	p. Arg294*	Nonsense	Severe	Serine protease	70
HB-5	c.880 C>T	Exon 8	p. Arg294*	Nonsense	Severe	Serine protease	70
HB-5.1	c.880 C>T	Exon 8	p. Arg294*	Nonsense	Severe	Serine protease	70
HB-5.2	c.880 C>T	Exon 8	p. Arg294*	Nonsense	Severe	Serine protease	70
HB-6	c.880 C>T	Exon 8	p. Arg294*	Nonsense	Severe	Serine protease	70
Carrier mother-7	c.1150 C>T	Exon 8	p. Arg384*	Nonsense	NA	Serine protease	5

*NA* not available

**Fig. 2:**
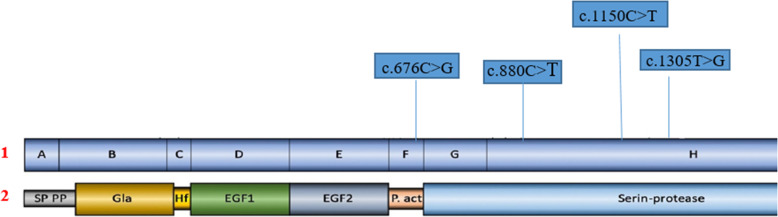
Distribution of variants in F9 in Egyptian patients. (1) Schematic representation of the F9 gene showing its 8 exons (A-H), with the location of the mutations found in the patients studied, (2) Factor IX protein; SP domain, signal peptide; PP, Pro peptide; Gla, gamma-carboxy-glutamic domain; EGF1 and EGF2, domains with homology to epidermal growth factors 1 and 2 respectively

HB patient 3 demonstrated another homozygous missense mutation (NM_000133.3:c.1305T>G, NP_000124.1: p.Cys435Trp) which is located in exon eight (Fig. 1b). One stop codon homozygous nonsense mutations located in exon 8 was characterized in cases and their affected family members within families no. 4, 5, and 6: (NM_000133.3:c.880C>T, NP_000124.1:p.Arg294*) (Fig. 1c). Another nonsense mutation presenting in a heterozygous state (NM_000133.3:c.1150C>T, NP_000124.1:p.Arg384*) was identified in exon 8 in carrier mother of Family 7 (Fig. 1d). All identified causative mutations within *F9* gene have been reported previously in *F9* Mutation Database sites and Human Genome Mutation Database HGMD [6] (Fig. 2).

## Discussion

Reviewing the literature, this is the first molecular HB study in Egypt. The pathogenic variants for HB were successfully identified in all studied patients; mutations were homozygous in patients and heterozygous in carrier mothers as per the X-linked recessive inheritance mode of the disease.

Mutational screening of *F9* gene in eleven clinically diagnosed Egyptian hemophilia-B patients and carrier mothers revealed four point mutations including two missense and two nonsense mutations that were correlating with phenotypic severity within the studied patients. Up to November 2018, the *F9* gene mutation database (EAHD Coagulation Factor Variant Databases) has recorded a total of 1094 mutations in 3713 HB patients [7], more than 3940 unique mutations have been reported so far in HGMD, 2020 [6]; point mutations accounts for 73.1% and mutations within the serine protease domain (SPD) account for about 56.1% among different populations [5, 8–17]. In agreement with that, all the detected mutations within our studied cohort were point mutations, three out of four detected mutations are within the protease domain, the greater part of codons (280–451) coded by the largest exon in the *F9* gene (exon 8).

Our patients presented with the classical phenotypic characteristics of the HB disease including severely affected males suffering from frequent spontaneous bleeding mainly in joints and muscles while boys with mild or moderate HB disease excessive bleeding only upon trauma.

We identified one missense mutation (NM_000133.3: c.676C>G; NP_000124.1: p.Arg226Gly) located in exon 6 in two HB patients (patients 1 and 2) who clinically presented with moderate phenotype. However, severe and moderate forms of the disease resulting from this mutation have been reported earlier in different populations like China, Turkey, and France [18–20]; this might be attributed to different ethnic backgrounds [5–21].

The missense mutation c.676C>G where cytosine was substituted to guanine at nucleotide 676 results in the replacement of positively polar arginine with uncharged polar amino acid glycine at codon 226 in activation peptide domain causing an alteration in its structure and decreased protein function, since arginine residues are known to be involved in the formation of salt bridges and hydrogen bonds [22].

Another identified missense mutation (NM_000133.3:c.1305T>G, NP_000124.1: p.Cys435Trp) was detected in one patient (patient 3) who presented with a mild phenotype. This mutation was previously reported in two Tunisian patients presenting with both severe and mild forms of the disease [21]. According to Elmahmoudi et al. 2012 [21], the discrepancy may be explained by the intervention of other hemostatic factors, which can modify the clinical severity of hemophilia. The corresponding mutation in codon 435 is located in the junction regions between the activation domain and its adjacent domains suggesting that the mutation might interfere with the normal cleavage process and resulting in the production of partially dysfunctional FIX proteins which might explain its association with the milder phenotype in our patient [5].

Interestingly, there are seventy patients’ data available in the Factor IX Variant database reporting the detected nonsense mutation (NM_000133.3:c.880C>T, NP_000124.1:p.Arg294*) in exon 8, leading mostly to moderate and severe forms of the disease in patients from different ethnic backgrounds. In the current study, the severe form of the disease was observed in three studied patients (patients 4, 5, and 6) and their affected families members where factor IX activity was <1% [5, 18, 23–26].

The seventh family (7) was seeking pre-conception counseling for a history of a deceased HB child. DNA from the carrier mother was sequenced and the causative mutation was characterized as a nonsense mutation (NM_000133.3:c.1150C>T, NP_000124.1:p.Arg384*) in exon 8 of the *F9* gene which was previously reported in Colombian, Brazilian, and Argentinian populations with moderate and severe forms of the disease [23, 27, 28]. The data obtained from this study will be used for carrier testing and prenatal diagnosis when requested.

The two nonsense mutations (p.Arg294*, p.Arg384*) were detected in eight patients descending from four unrelated HB families (families 4, 5, 6, 7) who presented with the severe phenotype, were previously described in patients bearing moderate and severe forms of the disease [5, 18, 23–26]. Our results are in agreement with the conclusion of Belvini et al. 2005 [26] who stated that the nonsense mutations are expected to produce truncated unstable proteins as a result of premature translation or the involvement of the Nonsense-Mediated mRNA Decay (NMD) system regardless of their location.

In addition, these two mutations (p.Arg294*, p.Arg384*) present in the serine protease domain; the greater part of which (codons 280–451) is coded by the largest exon in the *F9* gene (exon 8), where CpG dinucleotides are considered to be mutation hot spots that are the highly susceptible site for mutation within the *F9* gene [24–29].

Currently, the multiplex ligation dependent probe amplification (MLPA) method is used to detect large deletion or duplication mutations in F9 gene, however, it cannot detect point mutations that are considered as the major type of causative mutations in HB [8], and our studied patients were all diagnosed through direct sequencing of all coding exons of the *F9* gene.

## Conclusion

We could successfully define the mutational spectrum of *F9* gene in our cohort of eleven Egyptian HB patients descending from 6 unrelated pedigrees and one carrier female from a seventh family. A total of four different mutations were identified, which further confirms the high degree of heterogeneity in the mutations within *F9* gene. Mutation detection allows proper genetic counseling, carrier detection of female relatives, and prenatal genetic services.

Further studies with larger patients’ numbers are highly recommended to build an Egyptian database and confirm the genotype–phenotype correlation within our ethnic background. In absence of definitive curative treatment of hemophilia B disease, which results in most instances in patients’ mortality and/or disability, this database could help in trials for modern therapeutic possibilities where the molecular background has to be defined such as the evolving gene-editing technology.

## Data Availability

All data generated or analyzed during this study are included in this article.
